# Eating and lifestyle habits associated with regular soft drinks consumption among Brazilian adolescents: National Survey of School Health, 2019

**DOI:** 10.1590/1980-549720250007

**Published:** 2025-03-03

**Authors:** Carina Castelo Castelucci, Sanda Cristina Oancea, Luciana Bertoldi Nucci

**Affiliations:** I Pontifícia Universidade Católica de Campinas, School of Medicine, School of Life Sciences – Campinas (SP), Brazil.; II University of North Dakota, Public Health Program, Department of Population Health – Grand Forks, North Dakota, United States.; III Pontifícia Universidade Católica de Campinas, Graduate Program in Health Sciences, School of Life Sciences – Campinas (SP), Brazil.

**Keywords:** Health surveys, Adolescent behavior, Carbonated beverages, Adolescent, Health risk behaviors

## Abstract

**Objective:**

To assess the regular consumption of soft drinks among Brazilian adolescents according to sociodemographic characteristics, eating habits, and lifestyle.

**Methods:**

This is a cross-sectional study using data from 118,497 adolescents from the 2019 National Survey of School Health (PeNSE), a population survey periodically carried out in Brazil. The prevalence of regular soft drinks consumption was estimated and, using Poisson regression analysis, the association of this consumption with variables related to eating habits and lifestyle (physical and sedentary activities, use of cigarette and alcohol) was verified.

**Results:**

The frequency of regular soft drinks consumption was 17.2% (95%CI 16.6–17.8%). Multiple logistic regression analysis showed an association between regular soft drinks consumption and: living in the Southeast and Midwest regions (PR=1.49 and PR=1.50), boys (PR=1.22), eating meals while using a screen on five or more days a week (PR=1.20), eating breakfast less than five days a week (PR=1.14), consuming sweets five or more days a week (PR=2.16), and consuming fast food three or more times a week (PR=2.28). Spending more than three hours a day in sedentary activities (PR=1.18) and cigarette use (PR=1.22) and binge drinking (PR=1.21) were also statistically and significantly associated with regular soft drinks consumption.

**Conclusion:**

Adolescents’ regular consumption of soft drinks is associated with the region of residence, sex, and unhealthy eating and lifestyle habits. Interventions to promote the reduction of regular soft drinks consumption among Brazilian adolescents should consider innovative strategies that include comprehensive public policies appropriate to the profile of adolescents.

## INTRODUCTION

Adolescent health is a recurring topic in national^1,2^ and international^3,4^ population surveys. In this context, studies on excessive weight gain in this age group, which results in overweight or obesity among adolescents, stand out. These are conditions that can persist into adulthood, with consequences in the short, medium, and long term^5,6^, such as the development of noncommunicable diseases and injuries (NCDIs) and psychosocial impacts^7,8^.

In addition to overweight and obesity, other risk factors associated with adult NCDIs include sociodemographic characteristics, eating habits, and lifestyle^9^. Considering that adolescence is a period in which habits are established and often maintained in adulthood, the consolidation of healthy eating habits and lifestyles at this stage is essential for the prevention of overweight/obesity and, consequently, of NCDIs^10,11^.

Among unhealthy eating habits, soft drinks consumption has been investigated in both adolescents and adults due to the increasing consumption and association with NCDIs^12-14^. It is also known that excessive consumption of sweetened beverages contributes to the development of obesity, type 2 diabetes, cardiovascular diseases, and other metabolic conditions^15,16^.

The identification of characteristics and habits of adolescents in relation to the consumption of soft drinks can contribute to establishing prevention actions more directed to specific groups^16,17^. Thus, the objective of this study was to outline the profile of Brazilian adolescents in relation to soft drinks consumption and to verify possible associations of regular soft drinks consumption with sociodemographic characteristics, eating habits, and lifestyle.

## METHODS

### Study design and population

**Figure 1:**
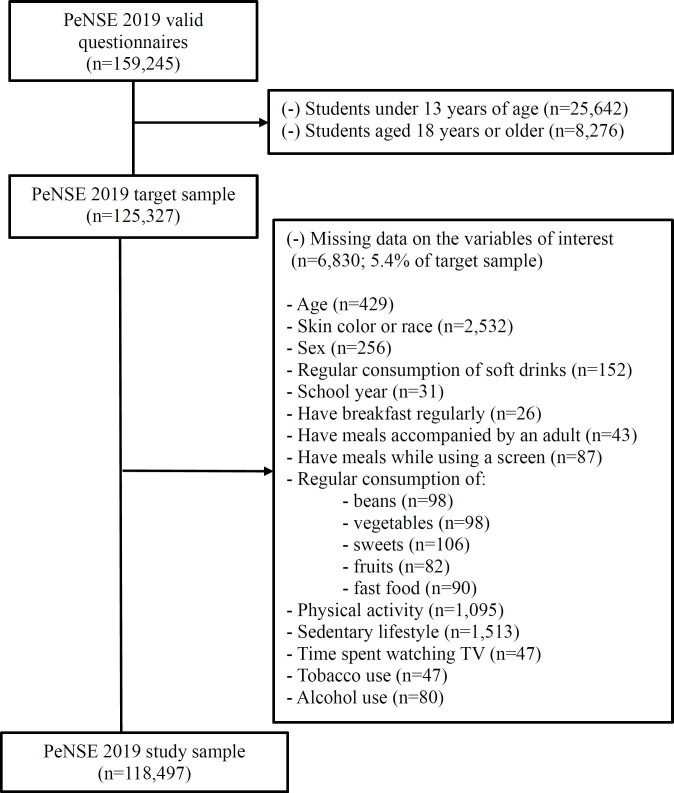
Sample selection flowchart.

This is a cross-sectional study in which microdata of the 2019 National Survey of School Health (*Pesquisa Nacional de Saúde do Escolar* – PeNSE) were analyzed. This school-based survey with national representativeness was carried out in 2009, 2012, 2015, and the most recent edition in 2019. The research consists of an electronic questionnaire self-administered by adolescents enrolled and regularly attending the seventh to ninth grades of Elementary School and the tenth to twelfth grades of High School (morning, afternoon, and evening shifts) from public and private schools. In the 2019 edition, data were collected from 4,253 schools in 1,288 municipalities and questionnaires from 160,721 students, totaling 159,245 valid questionnaires (in which the adolescent registered that he/she would like to participate in the research, in addition to informing sex and age, in classes that reached minimum requirements for achievement)^18^. In the present study, adolescents aged 13 to 17 years were included and those with missing data on the variables of interest were excluded. The flowchart of the exclusions made until obtaining the studied sample is described in Figure 1.

The 2019 edition of PeNSE was approved by the National Commission of Ethics in Research (*Comissão Nacional de Ética em Pesquisa* – Conep) of the National Health Council (*Conselho Nacional de Saúde* – CNS), under opinion No. 3.249.268, of April 8, 2019.

### Definition of the analyzed variables

The dependent variable under study was soft drink consumption, assessed through the question: "In the last seven days, in how many of them did you have soft drinks?". Responses were categorized into: sporadic consumption, defined as consumption in less than five days in the last week, and regular consumption, defined as consumption in five or more days in the last week^19^. The independent variables analyzed included sociodemographic characteristics, eating habits, and lifestyle.

The sociodemographic variables selected were region of residence (Northeast; North; Southeast; South; Midwest), sex (girls; boys), age group (13 to 15 years; 16 to 17 years), skin color or race (brown; white; Black; Asian; Indigenous), level of education (elementary school; high school), and type of school (public; private).

Questions about eating habits included: having meals accompanied by an adult, having meals while using a screen (TV, computer, or cell phone), and eating breakfast. Habit ("Do you usually…") and frequency (times a week) were considered. These variables were categorized into regular consumption, if consumed five or more days a week, or sporadic consumption, if consumed less than five days a week. Also regarding eating habits, questions were asked about the consumption in the last seven days of: beans, vegetables, sweets (exemplified in the question as: candies, confectionery, chocolates, gums, bonbons, lollipops, and others), fresh fruits or fruit salad. Regular consumption of these foods was defined as five or more days in the last seven days, or sporadic consumption, if it occurred less than five days in the last week^18,20^. Frequent fast food consumption was considered as three or more days, defined through the question: "In the last seven days, in how many of them did you eat in diners, hot dog stands, pizzerias, fast-food restaurants, etc.?". Less than three days was defined as sporadic consumption for this variable^21^.

Regarding lifestyle, the variables of physical and sedentary activities, use of cigarette and alcohol were evaluated. Physical activity was measured by total time in the last week, including time spent commuting, physical education classes, and leisure time. This total time was categorized as less than recommended (less than 300 minutes per week) or recommended (greater than or equal to 300 minutes per week)^22^. The daily time of sedentary activities (watching television, playing video games, using a cell phone or computer, and other sitting activities), without considering Saturday and Sunday and time spent sitting at school, was inquired regarding habit ("How many hours a day do you usually…?") and dichotomized as more than three hours or three hours or less per day^23^. Cigarette use was dichotomized into ‘yes’ for those who answered that they had smoked one or more cigarettes in the last 30 days and ‘no’ otherwise^24^. For the use of alcohol, no cutoff points were identified for the consumption of adolescents; therefore, the criteria for defining binge drinking (heavy episodic drinking) of the World Health Organization for adults was considered, categorizing the answers into ‘yes’ if the adolescent reported that he/she had four or more drinks on at least one occasion or ‘no’ if he/she had less than four drinks in the last 30 days^25^. The pieces of information on all analyzed variables were self-reported.

### Statistical analyses

Descriptive analyses were performed for all variables included in the study. Weighted prevalence and 95% confidence intervals (95%CI) were calculated for categorical variables. For comparisons between each exposure variable and the regular consumption of soft drinks, the Rao-Scott χ^2^ test was used, which considers the sample design of the study. A Poisson regression model was developed to verify possible associations between regular soft drinks consumption and independent variables. In the final model, only the variables that were statistically significant in the bivariate analysis were included. The analyses were carried out in SAS on Demand for Academics, version 3.81, considering the complex research sample design. A value of p<0.05 was considered statistically significant.

## RESULTS

The frequency of regular soft drinks consumption among adolescents was 17.3% (95%CI 16.7–18.0%). According to the bivariate analyses, there was an association between the prevalence of regular soft drinks consumption and the adolescent's region of residence, sex, and skin color/race. We observed a higher prevalence in boys (18.2%) when compared to girls (16.5%) (Table 1).

**Table 1:** Prevalence of soft drinks consumption by adolescents, according to sociodemographic variables. PeNSE, 2019.

		Soft drinks consumption*		p-value†
		Sporadic (n=99,421)	Regular (n=19,076)	
		% (95%CI)	% (95%CI)	
Region of residence				
	Northeast	87.7 (86.9–88.6)	12.3 (11.4–13.1)	<0.001
	North	87.0 (86.1–87.9)	13.0 (12.1–13.9)	
	Southeast	78.7 (77.3–80.1)	21.3 (19.9–22.7)	
	South	82.7 (81.4–84.0)	17.3 (15.9–18.6)	
	Midwest	78.7 (77.6–79.8)	21.3 (20.2–22.4)	
Sex				
	Girls	83.5 (82.7–84.2)	16.5 (15.8–17.3)	0.001
	Boys	81.8 (80.9–82.7)	18.2 (17.3–19.1)	
Age group (years)				
	13 to 15	82.9 (82.1–83.6)	17.1 (16.4–17.8)	0.389
	16 to 17	82.2 (81.0–83.5)	17.8 (16.5–19.0)	
Skin color or race				
	Brown	84.0 (83.25–84.8)	16.0 (15.2–16.8)	<0.001
	White	81.3 (80.3–82.2)	18.7 (17.8–19.7)	
	Black	82.0 (80.4–83.5)	18.0 (16.5–19.6)	
	Asian	81.8 (79.0–84.6)	18.2 (15.4–20.9)	
	Indigenous	84.0 (81.4–86.6)	16.0 (13.4–18.6)	
Level of education				
	Elementary school	82.5 (81.7–83.3)	17.5 (16.7–18.3)	0.671
	High school	82.8 (81.8–83.8)	17.2 (16.2–18.2)	
Type of school				
	Public	82.7 (81.9–83.4)	17.3 (16.6–18.0)	0.987
	Private	82.5 (81.7–83.3)	17.5 (16.7–18.3)	

PeNSE: National Survey of School Health; n: number of adolescents surveyed, without weighting; 95%CI: 95% confidence interval.

* Sporadic: <5 days in the last 7 days; Regular: ≥5 days in the last 7 days;

† p-value of Pearson's χ^2^ test with Rao-Scott correction.

In the unadjusted analysis of the variables of eating habits, a higher prevalence of regular consumption of soft drinks was observed among adolescents who eat meals while using screens (20.4%), those who have breakfast sporadically (20.2%), those who consume sweets (30.0%) regularly, and among adolescents who frequently consume fast food (39.2%) (Table 2).

**Table 2:** Prevalence of soft drinks consumption by adolescents, according to eating habits. PeNSE, 2019.

		Soft drinks consumption*		p-value†
		Sporadic (n=99,421)	Regular (n=19,076)	
		% (95%CI)	% (95%CI)	
Habit of having meals accompanied by an adult‡				
	Regular	82.9 (82.2–83.5)	17.1 (16.4–17.8)	0.144
	Sporadic	82.1 (81.1–83.1)	17.8 (16.8–18.9)	
Habit of having meals while using a screen‡				
	Sporadic	86.7 (85.9–87.5)	13.3 (12.5–14.1)	<0.001
	Regular	79.6 (78.7–80.5)	20.4 (19.5–21.3)	
Habit of eating breakfast‡				
	Regular	84.7 (84.0–85.4)	15.3 (14.6–16.0)	<0.001
	Sporadic	79.8 (78.8–80.7)	20.2 (19.3–21.2)	
Beans consumption*				
	Regular	83.0 (82.1–83.8)	17.0 (16.2–17.9)	0.171
	Sporadic	82.2 (81.3–83.1)	17.8 (16.9–18.6)	
Vegetables consumption*				
	Regular	82.8 (81.8–83.8)	17.2 (16.2–18.2)	0.723
	Sporadic	82.6 (81.9–83.3)	17.4 (16.7–18.1)	
Sweets consumption*				
	Sporadic	88.9 (88.3–89.4)	11.1 (10.5–11.6)	<0.001
	Regular	70.0 (68.7–71.2)	30.0 (28.7–31.3)	
Fruits consumption*				
	Sporadic	82.9 (82.2–83.6)	17.1 (16.4–17.8)	0.117
	Regular	82.0 (80.9–83.1)	18.0 (16.9–19.1)	
Fast food§				
	Sporadic	86.7 (86.1–87.3)	13.3 (12.7–13.9)	<0.001
	Frequent	60.8 (59.3–62.3)	39.2 (37.7–40.7)	

PeNSE: National Survey of School Health; n: number of adolescents surveyed, without weighting; 95%CI: 95% confidence interval.

* Sporadic: <5 days in the last 7 days; Regular: ≥5 days in the last 7 days;

† p-value of Pearson's χ^2^ test with Rao-Scott correction;

‡ Sporadic: <5 days; Regular: ≥5 days;

§ Sporadic: <3 days in the last 7 days; Frequent: ≥3 days in the last 7 days.

Regarding lifestyle, adolescents who reported more than three hours of sedentary activities per day had a higher prevalence of regular soft drinks consumption (20.5%). Cigarette and alcohol use were also associated with a higher prevalence of regular soft drinks consumption, with frequencies of 28.2 and 27.7%, respectively (Table 3).

**Table 3:** Prevalence of soft drinks consumption among adolescents, according to lifestyle. PeNSE, 2019.

		Soft drinks consumption*		p-value†
		Sporadic (n=99,421)	Regular (n=19,076)	
		% (95%CI)	% (95%CI)	
Total time of physical activity in the last week				
	Below recommended	82.8 (82.1–83.5)	17.2 (16.5–17.9)	0.273
	Recommended	82.2 (81.2–83.2)	17.8 (16.8–18.8)	
Daily time of sedentary activities (hours)				
	≤3	86.4 (85.7–87.1)	13.6 (12.9–14.3)	<0.001
	>3	79.5 (78.7–80.4)	20.5 (19.6–21.3)	
Cigarette use in the last 30 days				
	No	83.4 (82.8–84.0)	16.6 (16.0–17.2)	<0.001
	Yes	71.8 (69.4–74.2)	28.2 (25.8–30.6)	
Binge drinking				
	No	83.8 (83.1–84.4)	16.2 (15.6–16.9)	<0.001
	Yes	72.3 (70.5–74.1)	27.7 (25.9–29.5)	

PeNSE: National Survey of School Health; n: number of adolescents surveyed, without weighting; 95%CI: 95% confidence interval.

* Sporadic: <5 days in the last 7 days; Regular: ≥5 days in the last 7 days;

† p-value of Pearson's χ^2^ test with Rao-Scott correction.

In the multivariate analysis, there was a greater chance of regular consumption of soft drinks in adolescents living in the North (prevalence ratio — PR=1.13; 95%CI 1.04–1.23), Southeast (PR=1.49; 95%CI 1.40–1.60), South (PR=1.31; 95%CI 1.20–1.41), and Midwest (PR=1.50; 95%CI 1.41–1.59) when compared to Northeast residents. In addition, the regular consumption of soft drinks was more frequent among boys (PR= 1.22; 95%CI 1.16–1.29). As for eating habits, adolescents who reported having meals while using a screen had a greater chance of regular soft drinks consumption (PR=1.20; 95%CI 1.13–1.27). Likewise, those who reported eating breakfast sporadically (PR=1.14; 95%CI 1.08–1.21) and consuming sweets regularly (PR=2.16; 95%CI 2.03–2.29) also showed a greater chance of regular soft drinks consumption. Adolescents who reported consuming fast food frequently had 2.28 times the chance of having regular consumption of soft drinks when compared to those who consumed fast food sporadically (PR=2.28; 95%CI 2.16–2.41). Regular soft drinks consumption was 18% higher among adolescents with sedentary activities for more than three hours a day (PR=1.18; 95%CI 1.12–1.25). In addition, the regular consumption of soft drinks was more than 20% higher among those who reported using cigarettes (PR=1.22; 95%CI 1.11–1.33) and binge drinking (PR=1.21; 95%CI 1.12–1.30) (Table 4).

**Table 4:** Multiple logistic regression of the association between regular soft drinks consumption and sociodemographic, eating habits, and lifestyle variables. PeNSE, 2019.

		PR*(95%CI)	p-value†
Region of residence			
	Northeast	Ref.	
	North	1.13 (1.04–1.23)	0.004
	Southeast	1.49 (1.40–1.60)	<0.001
	South	1.31 (1.20–1.41)	<0.001
	Midwest	1.50 (1.41–1.59)	<0.001
Sex			
	Girls	Ref.	<0.001
	Boys	1.22 (1.16–1.29)	
Skin color or race			
	Brown	Ref.	
	White	1.05 (0.99–1.12)	0.088
	Black	1.03 (0.95–1.12)	0.449
	Asian	1.12 (0.97–1.29)	0.129
	Indigenous	1.01 (0.85–1.19)	0.934
Habit of having meals while using a screen‡			
	Sporadic	Ref.	<0.001
	Regular	1.20 (1.13–1.27)	
Habit of eating breakfast‡			
	Regular	Ref.	<0.001
	Sporadic	1.14 (1.08–1.21)	
Sweets consumption§			
	Sporadic	Ref.	<0.001
	Regular	2.16 (2.03–2.29)	
Fast food//			
	Sporadic	Ref.	<0.001
	Frequent	2.28 (2.16–2.41)	
Daily time of sedentary activities (hours)			
	≤3	Ref.	<0.001
	>3	1.18 (1.12–1.25)	
Cigarette use in the last 30 days			
	No	Ref.	<0.001
	Yes	1.22 (1.11–1.33)	
Binge drinking			
	No	Ref.	<0.001
	Yes	1.21 (1.12–1.30)	

PeNSE: National Survey of School Health; PR: prevalence ratio; 95%CI: 95% confidence interval; Ref.: reference category.

* PR adjusted for all variables present in the table;

† p-value of the logistic regression model;

‡ Sporadic: <5 days; Regular: ≥5 days;

§ Sporadic: <5 days in the last 7 days; Regular: ≥5 days in the last 7 days;

// Sporadic: <3 days in the last 7 days; Frequent: ≥3 days in the last 7 days.

## DISCUSSION

Our results point to the profile of regular soft drinks consumption among Brazilian adolescents, with regional variations, by skin color/race, and higher in boys. Regarding eating habits, the regular consumption of soft drinks was associated with eating meals while using screens, not having breakfast on five or more days of the week, and the frequent consumption of sweets and fast food. Lifestyles with prolonged time in sedentary activities, cigarette use, and binge drinking were also associated with regular soft drinks consumption.

The prevalence of regular soft drinks consumption observed in our study was much lower than the value of 42.8% (95%CI 32.4–50.7%) estimated in a global meta-analysis, which considered daily consumption^17^. However, when comparing our data on adolescents with data from other national surveys, we noticed a slightly higher prevalence. According to data from the 2017–2018 Consumer Expenditure Survey (*Pesquisa Nacional de Orçamentos Familiares* – POF), there is a prevalence of 15.4%^26^ in the total population, and authors of a study with data from the Surveillance System of Risk and Protective Factors for Noncommunicable Chronic Diseases by Telephone Survey (*Sistema de Vigilância de Fatores de Risco e Proteção para Doenças Crônicas por Inquérito Telefônico* – VIGITEL) identified a significant reduction in the frequency of regular consumption of soft drinks or synthetic juices by the population of state capitals and the Federal District, from 26.4% in 2008 to 15.0% in 2019^27^.

The regional variations found in our study point to a higher prevalence of regular consumption of soft drinks in adolescents living in the Southeast and Midwest regions, followed by the South and lower prevalence values in the North and Northeast. These prevalence values differ from the POF 2017–2018 results, in which the South region stands out with the highest regular consumption of soft drinks^26^. The higher prevalence of regular soft drinks consumption found for boys was also observed in other studies^12,16^.

In our study, a relevant finding was the positive association between regular consumption of soft drinks and eating habits markers of unhealthy diet, quite common in adolescents, such as having meals while using screens, skipping breakfast, and the consumption of sweets and fast food.

Excessive screen time is often studied in adolescents^28,29^, and eating meals while using screens can lead to loss of perception of food, interfering with physiological signs of hunger and satiety^29,30^. According to data from the Study of Cardiovascular Risks in Adolescents (*Estudo de Riscos Cardiovasculares em Adolescentes* – ERICA), approximately 60.0% of adolescents ate meals almost always or always in front of the television, a habit that was associated with the regular consumption of soft drinks in our study^30^. Authors of a research conducted in Chile showed that more than 85% of adolescents used screens during meals, consuming 42.3% of daily calories while watching TV. However, there were no significant differences in the nutrient profile between having meals with and without using screens, but higher weekly screen time was associated with a less healthy diet, which included higher consumption of sweetened beverages among Chilean adolescents^29^.

In a study on adolescents from the state of Espírito Santo, Brazil, sedentary behavior was associated with inadequate eating habits and poorer diet quality^31^. It is known that inadequate eating habits associated with sedentary lifestyle in childhood can trigger the onset of cardiometabolic diseases in the future^31,32^.

Alcohol use in adolescence tends to occur together with other health risk behaviors such as smoking^33^. When analyzing the data obtained in this study, we found an association between alcohol and tobacco use and regular soft drink consumption by adolescents. Authors of a longitudinal study with adolescents conducted in Finland observed that alcohol use in adolescence increases the risk of smoking in adulthood^34^. In Brazil, researchers of the ERICA study found that 21% of the interviewed adolescents had consumed alcohol in the 30 days prior to the interview^33^. Alcohol consumption by this population is worrisome due to the greater tendency to impulsivity in this age group, the impairment of brain development in childhood and adolescence caused by alcohol, and risk behaviors in the adolescent age group that may last into adulthood, influencing the development of other habits^35^.

Among the limitations of the present study, it should be noted that the obtained data were based on the adolescents’ reports, which may lead to information bias. Nonetheless, we emphasize that population surveys carried out in several countries also adopt this methodology to collect data in large samples^3,4,30^. Furthermore, we could not estimate the number of soft drinks consumed by the participants of the 2019 PeNSE due to the lack of information; however, the option for the variable of regular consumption of soft drinks proved to be adequate, as we analyzed adolescents’ habits.

Reducing the consumption of ultra-processed foods, including soft drinks, is one of the recommendations of the Dietary Guidelines for the Brazilian Population, given that they are associated with excessive calorie consumption and increased risk of obesity^36^. With our data, we emphasize the need for specific interventions, recognizing the profile of adolescents to promote healthier eating and lifestyle habits. These interventions should consider an integrated approach, acting not only in reducing the consumption of soft drinks, but also in factors associated with this regular consumption — such as the consumption of sweets and fast food. The National School Feeding Program (*Programa Nacional de Alimentação Escolar* – PNAE) serves public, philanthropic schools, and community entities in this sense, promoting healthy eating habits and developing initiatives on food and nutrition education^37^.

The incentive to have meals without using screens, to have breakfast daily, to reduce the time of sedentary activities, and to avoid the use of cigarette and alcohol requires, in addition to public policies, an awareness of these aspects by the family. These measures are paramount to protect the health of adolescents and reduce the burden of diseases related to the regular consumption of soft drinks.
