# Supplementary material for: Eating and lifestyle habits associated with regular soft drinks consumption among Brazilian adolescents: National Survey of School Health, 2019
Source: Rev Bras Epidemiol. 2025 Mar 3;28:e250007. doi: 10.1590/1980-549720250007 (PMC11884819; doi:10.1590/1980-549720250007)
Supplement: Translation

# Translation: Hábitos alimentares e de estilo de vida associados ao consumo regular de refrigerantes entre adolescentes brasileiros: Pesquisa Nacional de Saúde do Escolar (PeNSE), 2019

**DOI:** 10.1590/1980-549720250007.2

**Authors:** Carina Castelo Castelucci, Sanda Cristina Oancea, Luciana Bertoldi Nucci

**Affiliations:** I Pontifícia Universidade Católica de Campinas, Faculdade de Medicina, Escola de Ciências da Vida – Campinas (SP), Brasil.; II University of North Dakota, Public Health Program, Department of Population Health – Grand Forks, North Dakota, United States.; III Pontifícia Universidade Católica de Campinas, Programa de Pós-Graduação em Ciências da Saúde, Escola de Ciências da Vida – Campinas (SP), Brasil.

**Keywords:** Inquéritos epidemiológicos, Comportamento do adolescente, Refrigerantes, Adolescente, Comportamentos de risco à saúde

## Abstract

**Objetivo:**

Avaliar o consumo regular de refrigerantes em adolescentes brasileiros, de acordo com características sociodemográficas, de hábitos alimentares e de estilo de vida.

**Métodos:**

Estudo transversal que utilizou dados de 118.497 adolescentes da Pesquisa Nacional de Saúde do Escolar (PeNSE) de 2019. Foi estimada a prevalência do consumo regular de refrigerante e, utilizando regressão de Poisson, verificamos a associação desse consumo com variáveis relativas a hábitos alimentares e estilo de vida (atividades física e sedentária, uso de cigarro e álcool).

**Resultados:**

A frequência do consumo regular de refrigerante foi 17,2% (intervalo de confiança de 95% — IC95% 16,6–17,8%). Houve associação do consumo regular de refrigerantes com: morar nas Regiões Sudeste e Centro-Oeste (razão de prevalência — RP=1,49 e RP=1,50, respectivamente), ser do sexo masculino (RP=1,22), fazer refeições com uso de tela em cinco ou mais dias por semana (RP=1,20), ter o hábito de tomar café da manhã em menos de cinco dias por semana (RP=1,14), consumir guloseimas doces em cinco ou mais dias na semana (RP=2,16) e consumir *fast food* em três ou mais dias na semana (RP=2,28). Passar mais de três horas diárias em atividades sedentárias (RP=1,18), o uso de cigarro (RP=1,22) e *binge drinking* (RP=1,21) também apresentaram associação estatisticamente significativa com o consumo regular de refrigerantes.

**Conclusão:**

O consumo regular de refrigerantes por adolescentes está associado à região de residência, ao sexo e aos hábitos alimentares e de estilo de vida não saudáveis. As intervenções para promover a redução do consumo regular de refrigerantes entre os adolescentes brasileiros devem considerar estratégias inovadoras que incluam políticas públicas abrangentes adequadas ao perfil deles.

## INTRODUÇÃO

A saúde do adolescente é um tema recorrente em inquéritos populacionais nacionais^1,2^ e internacionais^3,4^. Nesse contexto, destacam-se estudos sobre o ganho excessivo de peso nesta faixa etária, que resulta em sobrepeso ou obesidade entre os adolescentes. Trata-se de condições que podem persistir na vida adulta, com consequências em curto, médio e longo prazos^5,6^, como o desenvolvimento de doenças e agravos não transmissíveis (DANT) e o impacto psicossocial^7,8^.

Além do sobrepeso e da obesidade, outros fatores de risco associados às DANT na vida adulta incluem características sociodemográficas, hábitos alimentares e estilo de vida^9^. Dado que a adolescência é um período em que hábitos são estabelecidos e frequentemente mantidos na vida adulta, a consolidação de hábitos alimentares e de estilos de vida saudáveis nessa fase é essencial para a prevenção do sobrepeso/obesidade e, consequentemente, das DANT^10,11^.

Entre os hábitos alimentares não saudáveis, o consumo de refrigerantes tem sido investigado tanto em adolescentes quanto em adultos em razão do crescente consumo e associação com as DANT^12-14^. Sabe-se também que o consumo excessivo de bebidas açucaradas contribui para o desenvolvimento de obesidade, diabetes tipo 2, doenças cardiovasculares e outras condições metabólicas^15,16^.

A identificação de características e hábitos dos adolescentes com relação ao consumo de refrigerantes pode contribuir para a definição de ações de prevenção mais direcionadas a grupos específicos^16,17^. Assim, o objetivo deste trabalho foi traçar o perfil dos adolescentes brasileiros com relação ao consumo de refrigerantes e verificar possíveis associações do consumo regular de refrigerantes com características sociodemográficas, hábitos alimentares e estilo de vida.

## MÉTODOS

### Delineamento e população do estudo

Trata-se de um estudo transversal em que foram analisados os microdados da Pesquisa Nacional de Saúde do Escolar (PeNSE) da edição de 2019. Este inquérito de base escolar com representatividade nacional foi realizado nos anos de 2009, 2012, 2015 e a edição mais recente, em 2019. A pesquisa consiste em um questionário eletrônico autopreenchido por adolescentes matriculados e que frequentam regularmente do sétimo ao nono ano do Ensino Fundamental e da primeira à terceira série do Ensino Médio (turnos matutino, vespertino e noturno) de escolas públicas e privadas. Na edição de 2019, foram coletados dados de 4.253 escolas de 1.288 municípios e questionários de 160.721 alunos, totalizando 159.245 questionários válidos (em que o adolescente registrou que gostaria de participar da pesquisa, além de informar o sexo e a idade, em turmas que atingiram requisitos mínimos para aproveitamento)^18^. Em nosso estudo, foram incluídos adolescentes de 13 a 17 anos e excluídos aqueles com dados faltantes nas variáveis de interesse. O fluxograma das exclusões realizadas até a obtenção da amostra estudada está descrito na Figura 1.

**Figura 1:** Fluxograma da seleção da amostra.

A edição de 2019 da PeNSE foi aprovada pela Comissão Nacional de Ética em Pesquisa (Conep) do Conselho Nacional de Saúde (CNS), com parecer n° 3.249.268, de 8 de abril de 2019.

### Definição das variáveis analisadas

A variável dependente em estudo foi o consumo de refrigerantes, avaliada por meio da pergunta: "Nos últimos sete dias, em quantos deles você tomou refrigerante?". As respostas foram categorizadas em: consumo esporádico, definido como o consumo em menos de cinco dias na última semana, e consumo regular, definido como o consumo em cinco ou mais dias na última semana^19^. As variáveis independentes analisadas compreendem características sociodemográficas, de hábitos alimentares e de estilo de vida.

As variáveis sociodemográficas selecionadas foram região de residência (Nordeste; Norte; Sudeste; Sul; Centro-Oeste), sexo (feminino; masculino), faixa etária (13 a 15 anos; 16 a 17 anos), cor ou raça (parda; branca; preta; amarela; indígena), escolaridade (ensino fundamental; ensino médio) e tipo de escola (pública; particular).

As questões sobre os hábitos alimentares incluíram: fazer a refeição acompanhado de um adulto, fazer a refeição com o uso de tela (TV, computador ou celular) e consumo de café da manhã. Foram considerados o hábito ("Você costuma…") e a frequência (vezes por semana). Essas variáveis foram categorizadas em consumo regular, se consumido em cinco ou mais dias na semana, ou consumo esporádico, se consumido em menos de cinco dias na semana. Ainda com relação aos hábitos alimentares, foram feitas questões sobre o consumo nos últimos sete dias de: feijão, legumes ou verduras, guloseimas doces (exemplificado na questão como: balas, confeitos, chocolates, chicletes, bombons, pirulitos e outros), frutas frescas ou salada de frutas. O consumo regular desses alimentos foi definido como cinco ou mais dias nos últimos sete dias, ou consumo esporádico, se ocorreu em menos de cinco dias na última semana^18,20^. O consumo frequente de *fast food* foi considerado como três ou mais dias, definido por meio da questão: "Nos últimos sete dias, em quantos deles você comeu em lanchonetes, barracas de cachorro-quente, pizzaria, *fast food* etc.?". Menos de três dias foi definido como consumo esporádico para essa variável^21^.

Com relação ao estilo de vida, foram avaliadas as variáveis de atividades físicas e sedentárias, uso de cigarro e álcool. A prática de atividade física foi medida pelo tempo total na última semana, incluindo o tempo gasto em deslocamento, aulas de educação física e no lazer. Esse tempo total foi categorizado em abaixo do recomendado (menos de 300 minutos por semana) ou recomendado (maior ou igual a 300 minutos por semana)^22^. O tempo diário de atividades sedentárias (assistir à televisão, jogar *videogame*, usar celular ou computador e outras atividades sentado), sem considerar sábado e domingo e tempo sentado na escola, foi perguntado com relação ao hábito ("Quantas horas por dia você costuma…?") e dicotomizado como mais de três horas ou três horas ou menos por dia^23^. O uso de cigarro foi dicotomizado em ‘sim’ para aqueles que responderam que fumaram um ou mais cigarros nos últimos 30 dias e ‘não’ caso contrário^24^. Para o uso de álcool, não foram identificados pontos de corte para o consumo de adolescentes, portanto foi considerado o critério para a definição de *binge drinking* (beber pesado episódico) da Organização Mundial da Saúde para adultos, categorizando-se as respostas em ‘sim’ se o adolescente relatou que consumiu quatro ou mais doses em pelo menos uma ocasião ou ‘não’ se consumiu menos de quatro doses nos últimos 30 dias^25^. As informações de todas as variáveis analisadas foram autorreferidas.

### Análises estatísticas

Foram realizadas análises descritivas para todas as variáveis incluídas no estudo. A prevalência ponderada e os intervalos de confiança de 95% (IC95%) foram calculados para as variáveis categóricas. Para comparações entre cada variável de exposição e o consumo regular de refrigerantes utilizamos o teste χ^2^ de Rao-Scott, que considera o delineamento amostral do estudo. Um modelo de regressão de Poisson foi elaborado para verificar possíveis associações entre o consumo regular de refrigerantes e as variáveis independentes. No modelo final foram incluídas apenas as variáveis que foram estatisticamente significativas na análise bivariada. As análises foram realizadas no SAS on Demand for Academics, versão 3.81, considerando-se o desenho amostral de pesquisa complexa. Foi considerado p<0,05 como estatisticamente significativo.

## RESULTADOS

A frequência do consumo regular de refrigerantes entre os adolescentes foi de 17,3% (IC95% 16,7–18,0%). As análises bivariadas mostraram que houve associação entre a prevalência de consumo regular de refrigerantes com a região de residência do adolescente, sexo e cor ou raça. Observou-se maior prevalência em adolescentes do sexo masculino (18,2%) quando comparados com as adolescentes do sexo feminino (16,5%) (Tabela 1).

**Tabela 1:** Prevalência do consumo de refrigerantes por adolescentes, de acordo com variáveis sociodemográficas. PeNSE, 2019.

		Consumo de refrigerantes*		p-valor†
		Esporádico (n=99.421)	Regular (n=19.076)	
		% (IC95%)	% (IC95%)	
Região de residência				
	Nordeste	87,7 (86,9–88,6)	12,3 (11,4–13,1)	<0,001
	Norte	87,0 (86,1–87,9)	13,0 (12,1–13,9)	
	Sudeste	78,7 (77,3–80,1)	21,3 (19,9–22,7)	
	Sul	82,7 (81,4–84,0)	17,3 (15,9–18,6)	
	Centro-Oeste	78,7 (77,6–79,8)	21,3 (20,2–22,4)	
Sexo				
	Feminino	83,5 (82,7–84,2)	16,5 (15,8–17,3)	0,001
	Masculino	81,8 (80,9–82,7)	18,2 (17,3–19,1)	
Faixa etária (anos)				
	13 a 15	82,9 (82,1–83,6)	17,1 (16,4–17,8)	0,389
	16 a 17	82,2 (81,0–83,5)	17,8 (16,5–19,0)	
Cor ou raça				
	Parda	84,0 (83,25–84,8)	16,0 (15,2–16,8)	<0,001
	Branca	81,3 (80,3–82,2)	18,7 (17,8–19,7)	
	Preta	82,0 (80,4–83,5)	18,0 (16,5–19,6)	
	Amarela	81,8 (79,0–84,6)	18,2 (15,4–20,9)	
	Indígena	84,0 (81,4–86,6)	16,0 (13,4–18,6)	
Escolaridade				
	Ensino fundamental	82,5 (81,7–83,3)	17,5 (16,7–18,3)	0,671
	Ensino médio	82,8 (81,8–83,8)	17,2 (16,2–18,2)	
Tipo de escola				
	Pública	82,7 (81,9–83,4)	17,3 (16,6–18,0)	0,987
	Particular	82,5 (81,7–83,3)	17,5 (16,7–18,3)	

PeNSE: Pesquisa Nacional de Saúde do Escolar; n: número de adolescentes estudados, sem ponderação; IC95%: intervalo de confiança de 95%.

* Esporádico: <5 dias nos últimos 7 dias; Regular: ≥5 dias nos últimos 7 dias;

† p-valor do teste χ^2^ de Pearson com correção de Rao-Scott.

Na análise não ajustada das variáveis de hábitos alimentares foi observada maior prevalência de consumo regular de refrigerantes entre os adolescentes que fazem as refeições com o uso de tela (20,4%), aqueles que consomem café da manhã esporadicamente (20,2%), aqueles que consomem guloseimas doces (30,0%) regularmente e entre os adolescentes que consomem *fast food* frequentemente (39,2%) (Tabela 2).

**Tabela 2:** Prevalência do consumo de refrigerantes por adolescentes, de acordo com hábitos alimentares. PeNSE, 2019.

		Consumo de refrigerantes*		p-valor†
		Esporádico (n=99.421)	Regular (n=19.076)	
		% (IC95%)	% (IC95%)	
Hábito de fazer refeição acompanhada de um adulto‡				
	Regular	82,9 (82,2–83,5)	17,1 (16,4–17,8)	0,144
	Esporádico	82,1 (81,1–83,1)	17,8 (16,8–18,9)	
Hábito de fazer a refeição com uso de tela‡				
	Esporádico	86,7 (85,9–87,5)	13,3 (12,5–14,1)	<0,001
	Regular	79,6 (78,7–80,5)	20,4 (19,5–21,3)	
Hábito de tomar café da manhã‡				
	Regular	84,7 (84,0–85,4)	15,3 (14,6–16,0)	<0,001
	Esporádico	79,8 (78,8–80,7)	20,2 (19,3–21,2)	
Consumo de feijão*				
	Regular	83,0 (82,1–83,8)	17,0 (16,2–17,9)	0,171
	Esporádico	82,2 (81,3–83,1)	17,8 (16,9–18,6)	
Consumo de legumes ou verduras*				
	Regular	82,8 (81,8–83,8)	17,2 (16,2–18,2)	0,723
	Esporádico	82,6 (81,9–83,3)	17,4 (16,7–18,1)	
Consumo de guloseimas doces*				
	Esporádico	88,9 (88,3–89,4)	11,1 (10,5–11,6)	<0,001
	Regular	70,0 (68,7–71,2)	30,0 (28,7–31,3)	
Consumo de frutas*				
	Esporádico	82,9 (82,2–83,6)	17,1 (16,4–17,8)	0,117
	Regular	82,0 (80,9–83,1)	18,0 (16,9–19,1)	
*Fast food* §				
	Esporádico	86,7 (86,1–87,3)	13,3 (12,7–13,9)	<0,001
	Frequente	60,8 (59,3–62,3)	39,2 (37,7–40,7)	

PeNSE: Pesquisa Nacional de Saúde do Escolar; n: número de adolescentes estudados, sem ponderação; IC95%: intervalo de confiança de 95%.

* Esporádico: <5 dias nos últimos 7 dias; Regular: ≥5 dias nos últimos 7 dias;

† p-valor do teste χ^2^ de Pearson com correção de Rao-Scott;

‡ Esporádico: <5 dias; Regular: ≥5 dias;

§ Esporádico: <3 dias nos últimos 7 dias; Frequente: ≥3 dias nos últimos 7 dias.

Com relação ao estilo de vida, os adolescentes que relataram tempo de atividades sedentárias superior a três horas por dia apresentaram maior prevalência de consumo regular de refrigerantes (20,5%). O uso de cigarro e álcool também foram associados a uma maior prevalência no consumo regular de refrigerantes, com frequências de 28,2 e 27,7%, respectivamente (Tabela 3).

**Tabela 3:** Prevalência do consumo de refrigerantes em adolescentes, de acordo com o estilo de vida. PeNSE, 2019.

		Consumo de refrigerantes*		p-valor†
		Esporádico (n=99.421)	Regular (n=19.076)	
		% (IC95%)	% (IC95%)	
Tempo total de atividade física na última semana				
	Abaixo do recomendado	82,8 (82,1–83,5)	17,2 (16,5–17,9)	0,273
	Recomendado	82,2 (81,2–83,2)	17,8 (16,8–18,8)	
Tempo diário de atividades sedentárias (horas)				
	3 ou menos	86,4 (85,7–87,1)	13,6 (12,9–14,3)	<0,001
	>3	79,5 (78,7–80,4)	20,5 (19,6–21,3)	
Uso de cigarro nos últimos 30 dias				
	Não	83,4 (82,8–84,0)	16,6 (16,0–17,2)	<0,001
	Sim	71,8 (69,4–74,2)	28,2 (25,8–30,6)	
*Binge drinking*				
	Não	83,8 (83,1–84,4)	16,2 (15,6–16,9)	<0,001
	Sim	72,3 (70,5–74,1)	27,7 (25,9–29,5)	

PeNSE: Pesquisa Nacional de Saúde do Escolar; n: número de adolescentes estudados, sem ponderação; IC95%: intervalo de confiança de 95%.

* Esporádico: <5 dias nos últimos 7 dias; Regular: ≥5 dias nos últimos 7 dias;

† p-valor do teste χ^2^ de Pearson com correção de Rao-Scott.

Na análise multivariada, observou-se maior chance de consumo regular de refrigerantes nos adolescentes moradores das Regiões Norte (razão de prevalência —RP=1,13; IC95% 1,04–1,23), Sudeste (RP=1,49; IC95% 1,40–1,60), Sul (RP=1,31; IC95% 1,20–1,41) e Centro-Oeste (RP=1,50; IC95% 1,41–1,59) quando comparados aos moradores do nordeste. Além disso, o consumo regular de refrigerantes foi mais frequente entre os adolescentes do sexo masculino (RP=1,22; IC95% 1,16–1,29). Com relação aos hábitos alimentares, os adolescentes que relataram fazer refeição com uso de tela apresentaram maior chance de consumo regular de refrigerantes (RP=1,20; IC95% 1,13–1,27). Do mesmo modo, aqueles que responderam tomar café da manhã esporadicamente (RP=1,14; IC95% 1,08–1,21) e consumir guloseimas doces regularmente (RP=2,16; IC95% 2,03–2,29) também mostraram maior chance de consumo regular de refrigerantes. Os adolescentes que relataram consumir *fast food* frequentemente tiveram 2,28 vezes a chance de ter consumo regular de refrigerantes quando comparados aos que consumiram *fast food* esporadicamente (RP=2,28; IC95% 2,16–2,41). O consumo regular de refrigerantes foi 18% maior entre adolescentes com atividades sedentárias em mais de três horas por dia (RP=1,18; IC95% 1,12–1,25). Além disso, o consumo regular de refrigerantes foi mais de 20% maior entre aqueles que relataram o uso de cigarro (RP=1,22; IC95% 1,11–1,33) e *binge drinking* (RP=1,21; IC95% 1,12–1,30) (Tabela 4).

**Tabela 4:** Regressão logística múltipla da associação entre o consumo regular de refrigerantes e variáveis sociodemográficas, de hábitos alimentares e de estilo de vida. PeNSE, 2019.

		RP*(IC95%)	p-valor†
Região de residência			
	Nordeste	Ref.	
	Norte	1,13 (1,04–1,23)	0,004
	Sudeste	1,49 (1,40–1,60)	<0,001
	Sul	1,31 (1,20–1,41)	<0,001
	Centro-Oeste	1,50 (1,41–1,59)	<0,001
Sexo			
	Feminino	Ref.	<0,001
	Masculino	1,22 (1,16–1,29)	
Cor ou raça			
	Parda	Ref.	
	Branca	1,05 (0,99–1,12)	0,088
	Preta	1,03 (0,95–1,12)	0,449
	Amarela	1,12 (0,97–1,29)	0,129
	Indígena	1,01 (0,85–1,19)	0,934
Hábito de fazer a refeição com o uso de tela‡			
	Esporádico	Ref.	<0,001
	Regular	1,20 (1,13–1,27)	
Hábito de tomar café da manhã‡			
	Regular	Ref.	<0,001
	Esporádico	1,14 (1,08–1,21)	
Consumo de guloseimas doces§			
	Esporádico	Ref.	<0,001
	Regular	2,16 (2,03–2,29)	
*Fast food* //			
	Esporádico	Ref.	<0,001
	Frequente	2,28 (2,16–2,41)	
Tempo diário de atividades sedentárias (horas)			
	3 ou menos	Ref.	<0,001
	>3	1,18 (1,12–1,25)	
Uso de cigarro nos últimos 30 dias			
	Não	Ref.	<0,001
	Sim	1,22 (1,11–1,33)	
*Binge drinking*			
	Não	Ref.	<0,001
	Sim	1,21 (1,12–1,30)	

PeNSE: Pesquisa Nacional de Saúde do Escolar; RP: razão de prevalências; IC95%: intervalo de confiança de 95%; Ref.: categoria de referência.

* RP ajustada para todas as variáveis presentes na tabela;

† p-valor do modelo de regressão logística;

‡ Esporádico: <5 dias; Regular: ≥5 dias;

§ Esporádico: <5 dias nos últimos 7 dias; Regular: ≥5 dias nos últimos 7 dias;

// Esporádico: <3 dias nos últimos 7 dias; Frequente: ≥3 dias nos últimos 7 dias.

## DISCUSSÃO

Os resultados do presente estudo apontam o perfil de consumo regular de refrigerantes entre adolescentes brasileiros, com variações regionais, por cor ou raça e maior nos adolescentes do sexo masculino. Com relação aos hábitos alimentares, o consumo regular de refrigerantes foi associado ao uso de telas durante as refeições, à omissão do café da manhã em cinco ou mais dias da semana e ao consumo frequente de guloseimas doces e *fast food*. Estilos de vida com tempo prolongado em atividades sedentárias, uso de cigarro e *binge drinking* também foram associados ao consumo regular de refrigerantes.

A prevalência do consumo regular de refrigerantes observada em nosso estudo foi bastante inferior ao valor de 42,8% (IC95% 32,4–50,7%) estimado em metanálise global, que considerou o consumo diário^17^. No entanto, comparando-se nossos dados de adolescentes com dados de outros inquéritos nacionais, notamos uma prevalência sutilmente superior. Dados da Pesquisa Nacional de Orçamentos Familiares (POF) de 2017–2018 mostram prevalência de 15,4%^26^ na população total, e um estudo com dados do Sistema de Vigilância de Fatores de Risco e Proteção para Doenças Crônicas por Inquérito Telefônico (VIGITEL) identificou redução expressiva na frequência de consumo regular de refrigerantes ou sucos artificiais pela população das capitais estaduais e do Distrito Federal, de 26,4% em 2008 para 15,0% em 2019^27^.

As variações regionais encontradas em nosso estudo apontam para maior prevalência do consumo regular de refrigerantes nos adolescentes residentes nas Regiões Sudeste e Centro-Oeste, seguidas pela Região Sul e menores prevalências no norte e nordeste. Essas prevalências diferem dos resultados da POF 2017–2018, em que a Região Sul se destaca com o maior consumo regular de refrigerantes^26^. A maior prevalência de consumo regular de refrigerantes encontrada para os adolescentes do sexo masculino também foi observada em outros estudos^12,16^.

Neste trabalho, achado relevante foi a associação positiva entre consumo regular de refrigerantes e hábitos alimentares marcadores de alimentação não saudável, bastante comuns em adolescentes, como fazer a refeição com o uso de tela, a omissão do café da manhã e o consumo de guloseimas doces e *fast food*.

O tempo excessivo de uso de telas é frequentemente estudado em adolescentes^28,29^, e o uso de telas durante as refeições pode acarretar perda da percepção da alimentação, interferindo nos sinais fisiológicos de fome e saciedade^29,30^. Dados do estudo ERICA mostraram que aproximadamente 60,0% dos adolescentes realizavam as refeições quase sempre ou sempre em frente à televisão, hábito que mostrou associação com o consumo regular de refrigerantes em nosso estudo^30^. Pesquisa realizada no Chile mostrou que mais de 85% dos adolescentes usavam telas durante as refeições, consumindo 42,3% das calorias diárias enquanto assistiam à TV. No entanto, não houve diferenças significativas no perfil de nutrientes entre refeições com e sem telas, mas o maior tempo de tela semanal foi associado a uma dieta menos saudável, que incluiu maior consumo de bebidas adoçadas dos adolescentes chilenos^29^.

Em um estudo com adolescentes do Espírito Santo, o comportamento sedentário foi associado a hábitos alimentares inadequados e pior qualidade da dieta^31^. Sabe-se que hábitos alimentares inadequados associados ao sedentarismo na infância podem desencadear o aparecimento de doenças cardiometabólicas no futuro^31,32^.

O uso de álcool na adolescência tende a ocorrer em conjunto com outros comportamentos de risco à saúde, como o tabagismo^33^. A análise dos dados obtidos neste estudo mostra associação de consumo de álcool, tabaco e consumo regular de refrigerante por adolescentes. Estudo longitudinal com adolescentes realizado na Finlândia observou que o uso de álcool na adolescência aumenta o risco de tabagismo na vida adulta^34^. No Brasil, o ERICA detectou que 21% dos adolescentes entrevistados haviam consumido bebida alcoólica nos 30 dias anteriores à entrevista^33^. O consumo de álcool por esse grupo é preocupante pela maior tendência à impulsividade nessa faixa etária, pelo prejuízo ao desenvolvimento cerebral na infância e na adolescência causado pelo álcool e por comportamentos de risco na faixa etária da adolescência poderem perdurar na vida adulta, influenciando no desenvolvimento de outros hábitos^35^.

Entre as limitações do presente estudo, deve-se destacar que os dados obtidos se basearam no relato dos adolescentes, podendo acarretar viés de informação. No entanto, ressaltamos que inquéritos populacionais realizados em vários países também adotam essa metodologia para coletar dados em grandes amostras^3,4,30^. Além disso, não foi possível estimar a quantidade de refrigerante consumida pelos participantes da PeNSE de 2019 por conta da inexistência de informações, no entanto a opção pela variável de consumo regular de refrigerantes se mostrou adequada, visto que analisamos os hábitos dos adolescentes.

A redução do consumo de alimentos ultraprocessados, incluindo o de refrigerantes, é uma das recomendações do *Guia Alimentar para a População Brasileira*, tendo em vista que eles estão associados ao consumo excessivo de calorias e ao maior de risco de obesidade^36^. Os dados do nosso estudo ressaltam a necessidade de intervenções específicas, reconhecendo o perfil dos adolescentes para promover hábitos alimentares e de estilo de vida mais saudáveis. Essas intervenções devem considerar uma abordagem integrada, atuando não apenas na redução de consumo de refrigerantes, mas também nos fatores associados a esse consumo regular, como o consumo de guloseimas doces e *fast food*. O Programa Nacional de Alimentação Escolar (PNAE) atua nas escolas públicas, filantrópicas e em entidades comunitárias nesse sentido, promovendo hábitos alimentares saudáveis e o desenvolvimento de ações de educação alimentar e nutricional^37^.

O incentivo para fazer as refeições sem o uso de tela, tomar café da manhã diariamente, reduzir o tempo de atividades sedentárias e evitar o uso de cigarro e álcool exige, além de políticas públicas, uma conscientização da família para esses aspectos. Essas medidas são essenciais para proteger a saúde dos adolescentes e reduzir o ônus das doenças relacionadas ao consumo regular de refrigerantes.
